# Immunomagnetic reduction detects amyloid β 1–42, but neither *x*–42 nor 1–*y*

**DOI:** 10.1039/d6ra01450j

**Published:** 2026-07-02

**Authors:** Huei-Chun Liu, Chin-Yi Lin, Chia-Shin Ho, Jui-Feng Chang, Kun-Hung Lee, Shieh-Yueh Yang

**Affiliations:** a MagQu Co., Ltd New Taipei 231 Taiwan syyang@magqu.com

## Abstract

Amyloid β 1–42 peptide (Aβ_1–42_) is listed in the diagnostic guidelines as a plasma analyte for evaluating amyloid neuropathology in Alzheimer's disease. Owing to its ultralow concentrations in plasma, ultrasensitive analytical technologies have been developed for its accurate detection. Immunomagnetic reduction (IMR) is an ultrasensitive assay. In contrast to conventional immunoassays, which involve pairs of primary antibodies against the C-terminal and N-terminal domains of Aβ_1–42_ for achieving high specificity, IMR utilizes a single primary antibody against the C-terminal domain of Aβ_1–42_. Thus, IMR may be used to assay not only Aβ_1–42_ but also other Aβ peptides, such as Aβ_*x*–42_. In this work, the specificity of assaying Aβ_1–42_ using IMR was investigated. The binding specificities between the C-terminal antibody used in the IMR reagent and Aβ peptides were examined through western blot analysis. Furthermore, the interference effects of other Aβs on Aβ_1–42_ quantification using IMR were explored. The analytical performance, including the analytical curve, low limit of detection, high limit of detection, and storage stability of the reagents, was investigated. The C-terminal antibody only binds to Aβ_1–42_, and not to Aβ_3–42_, pyroglutamate-modulated Aβ_3–42_ (Aβ_p3–42_), Aβ_1–55_, Aβ_1–38_, Aβ_3–40,_ or Aβ_1–40_. The IMR Aβ_1–42_ assay developed using this antibody also demonstrated high selectivity for Aβ_1–42_. The lower and upper limits of detection for assaying Aβ_1–42_ were 17 fg mL^−1^ and 30 000 pg mL^−1^, respectively. Furthermore, the storage stability of the IMR Aβ_1–42_ reagent at 2–8 °C was at least 440 days. These results reveal that the IMR assay of Aβ_1–42_ exhibits high sensitivity and high specificity enabling precise measurement of Aβ_1–42_ concentrations in human plasma.

## Abstract 2

IMR assay shows the high specificity in detecting amyloid β 1–42 peptide (Aβ_1–42_) with the existence of other Aβ peptides.

## Graphical abstract

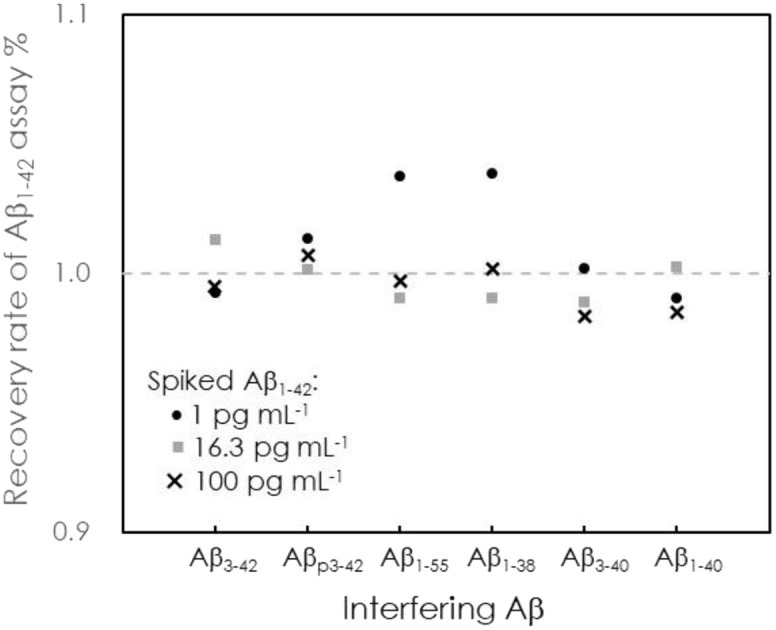

## Introduction

The revised criteria for the diagnosis and staging of Alzheimer's disease using human plasma analytes suggest the use of the amyloid β 1–42 peptide (Aβ_1–42_) or phosphorylated tau protein (pTau) as a plasma analyte to confirm Alzheimer's pathophysiology and total tau protein (T-Tau) as a biomarker to confirm Alzheimer's disease in patients.^1^ These pathological biomarkers are present in human plasma at ultralow concentrations (*e.g.*, pg mL^−1^). To precisely detect abnormalities in these plasma biomarkers, ultrasensitive detection is needed in clinical practice.

Immunomagnetic reduction (IMR) is an ultrasensitive technology that can detect biomolecules at concentrations of fg mL^−1^-to-pg mL^−1^.^2,3^ Since 2012, extensive studies and clinical trials have validated the application of IMR for assessing amnestic mild cognitive impairment (aMCI) and early-stage Alzheimer's disease dementia (eADD) through detecting plasma Aβ_1–42_, T-Tau and pTau levels.^4–8^ Hence, IMR assays have been approved for clinical use in Taiwan and Europe.

Aβ peptides are generated from the amyloid precursor protein through cleavage by β- and γ-secretases.^9^ Several Aβ species have been identified, distinguished mainly by the differences in number of amino acid residues.^10^ These species are further grouped according to N-terminal pyroglutamate modifications or aggregation properties.^11,12^ Among these species, Aβ_1–42_, especially aggregated Aβ_1–42_, plays a role in the amyloidosis cascade hypothesis of Alzheimer's disease.^13,14^

In sandwich immunoassays, two kinds of antibodies are used for a target biomolecule. In the case of measuring Aβ_1–42_, one antibody (the capture antibody) is against the C-terminal domain (*x*–42) of Aβ_1–42_, and the other antibody (the labeling antibody) is against the N-terminal domain (1–*y*) of Aβ_1–42_. Thus, the sandwich immunoassay is able to specifically measure Aβ_1–42_, and not Aβ_*x*–42_ or Aβ_1–*y*_.

In contrast to sandwich technologies, only the capture antibody that is immobilized on magnetic nanoparticles is used in the IMR assay. When antibody-functionalized magnetic nanoparticles bind to target biomolecules, the resultant magnetic susceptibility of the nanoparticles dispersed in a phosphate-buffered saline (PBS) solution decreases.^15,16^ This reduction in the magnetic susceptibility of the IMR reagent is sensed and then converted into the concentration of target biomolecules *via* a standard curve, allowing quantification of the biomarkers of interest.^17^ In the case of the IMR Aβ_1–42_ assay, the antibody is against the C-terminal domain, which contains amino acid residues 37–42. Thus, the antibody can bind Aβ_1–42_ as well as potentially other Aβ species containing 37–42 amino acid residues, such as Aβ_*x*–42_. Consequently, the measured concentrations may not be specific to Aβ_1–42_, and could also reflect the presence of other Aβ peptides. It is worth investigating the specificity of the Aβ_1–42_ measurement with IMR.

In this work, the binding specificities between the C-terminal antibody and Aβ peptides were examined through western blot analysis. Furthermore, the interference effects of other Aβ peptides on Aβ_1–42_ were explored using IMR. Analytical performance, including analytical curves, lower and upper limits of detection, and the storage stability of the reagents, was investigated.

## Materials and methods

### Preparation of the antibody

Monoclonal antibodies were produced using the mouse ascites method in BALB/c mice. The mice were primed through intraperitoneal injection of pristane (2,6,10,14-tetramethylpentadecane). One week later, hybridoma cells secreting the target monoclonal antibody were inoculated intraperitoneally. Ascitic fluid was collected upon visible abdominal distension and clarified through centrifugation to remove cellular debris. The monoclonal antibody was subsequently purified from the ascitic fluid using protein A affinity chromatography. The purified antibody was concentrated and diluted in phosphate-buffered saline (PBS) before being stored at −80 °C until further use. All animal experimentation procedures were conducted in accordance with the “Guidelines for the Management and Use of Laboratory Animals” issued by the Ministry of Agriculture, Taiwan and were approved by the Institutional Animal Care and Use Committee (IACUC) of MagQu.

### Western blot

Recombinant or synthetic Aβ peptides (160 ng), including full-length Aβ_1–42_ (A1075; Sigma), Aβ_3–42_ (AS-63715; Anaspec), pyroglutamate-modulated Aβ_3–42_ (Aβ_p3–42_) (AS-29907; Anaspec), Aβ_1–55_ (AS-63335; Anaspec), Aβ_1–38_ (AS-24333; Anaspec), Aβ_3–40_ (AS-61029; Anaspec) and Aβ_1–40_ (A9810; Sigma), were prepared in equal amounts for western blot analysis. These proteins were separated by 10% sodium dodecyl sulfate–polyacrylamide gel electrophoresis (SDS–PAGE) under reducing conditions and transferred to a polyvinylidene fluoride (PVDF) membrane. The membranes were incubated with a mouse anti-Aβ_1–42_ primary antibody (AT-AB2-12F2; MagQu) at a dilution of 1 : 1000, followed by incubation with alkaline phosphatase–conjugated secondary antibodies at a dilution of 1 : 5000. Signal development was performed using 5-bromo-4-chloro-3-indolyl-phosphate (BCIP).

### ELISA assay

An enzyme-linked immunosorbent assay (ELISA) was performed to evaluate antibody binding. Briefly, 100 ng of antigen per well was diluted in phosphate-buffered saline (PBS) and coated onto a 96-well ELISA plate (100 µL per well) by overnight incubation at 4 °C. The wells were subsequently washed twice with PBST (PBS containing Tween-20) and blocked with 200 µL of blocking buffer at room temperature for 1 h. After blocking, the wells were washed three times with PBST and incubated with serially diluted primary antibodies (1 : 1000 to 1 : 80 000; 100 µL per well) at room temperature for 60 min. Following three washes with PBST, a secondary antibody diluted 1 : 10 000 (100 µL per well) was added and incubated for 60 min at room temperature. The wells were then washed three times, and 100 µL of tetramethylbenzidine (TMB) substrate was added for color development for 15–30 minutes. The reaction was terminated by adding 100 µL of 1 N HCl, and absorbance was measured at 450 nm using an ELISA reader (Synergy HT, Biotek).

### Preparation of the IMR reagent

The processes to synthesize magnetic Fe_3_O_4_ nanoparticles are described elsewhere in detail (ref. 18 and 19). A solution consisting of a stoichiometric ratio of 1 : 2 ferrous sulfate heptahydrate and ferric chloride hexahydrate was combined with an equal volume of aqueous dextran. The mixture was heated and titrated with a base solution to form black Fe_3_O_4_ particles. Reagents with the desired magnetic concentration were obtained by diluting the concentrated magnetic fluid with a pH 7.4 PBS solution. The mouse anti-Aβ_1–42_ primary antibody (AT-AB2-12F2; MagQu) was covalently bound to dextran on the particle surface. Through magnetic separation, the unbound antibodies were separated from the magnetic solution such that the IMR reagent could be used to assay Aβ_1–42_.

### IMR measurements

For the IMR Aβ_1–42_ assay, 60 µL of reagent (MF-AB2-0060B; MagQu) was mixed with 60 µL of sample. The real-time alternative-current magnetic susceptibility of the reagent was recorded with an analyzer (XacPro-S; MagQu) during the associations between the target biomolecules and antibody-functionalized magnetic nanoparticles. During the associations, it is not necessary to perform any steps, such as washing away unbound antibody-functionalized nanoparticles. Hence, IMR is a wash-free immunoassay. Furthermore, the detected signals in IMR are magnetic, not optical as in conventional immunoassays. The detected optical signals are potentially interfered with by colorful chemicals/biomolecules such as hemoglobin, bilirubin, and intralipid.^20,21^ In IMR, which detects magnetic signals, color interference does not matter. The variations in IMR measurements are suppressed as compared with optical-based immunoassays.^20,21^ In this work, duplicated measurements were performed for each sample. The average values of the duplicate concentrations are reported.

## Results

### Specificity of the anti-Aβ_1–42_ antibody

**Fig. 1:**
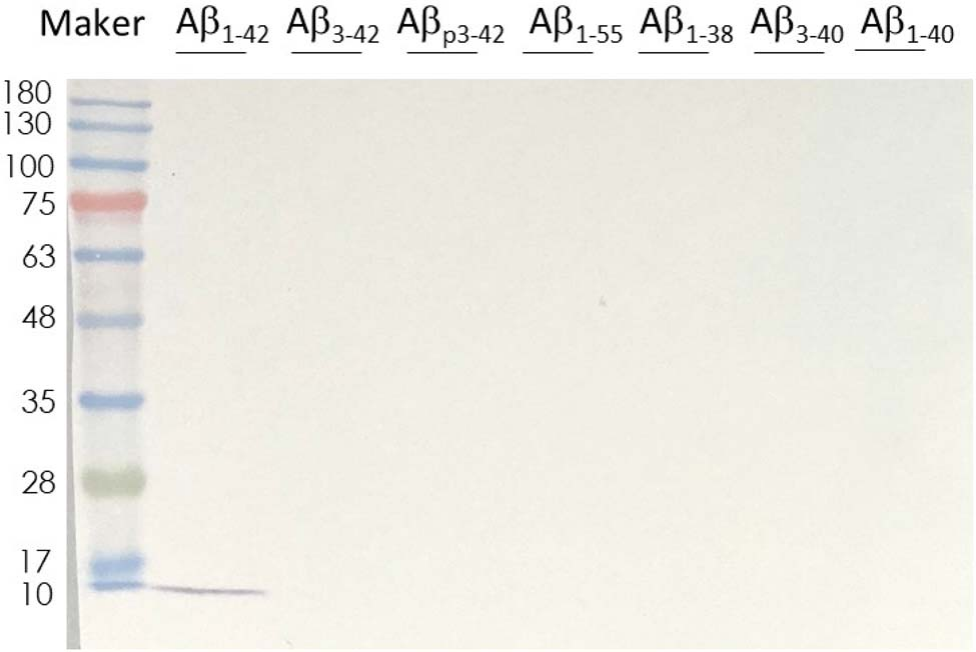
Western blot image of the C-terminal anti-Aβ_1–42_ antibody against Aβ_1–4_, Aβ_3–42_, pyroglutamate-modulated Aβ_3–42_ (Aβ_p3–42_), Aβ_1–55_, Aβ_1–38_, Aβ_3–40,_ and Aβ_1–40_ spiked in phosphate-buffered saline (PBS) solutions.

The specificity of the mouse anti-Aβ_1–42_ primary antibody (AT-AB2-12F2, MagQu) used in the IMR reagent was investigated through western blot analysis, as shown in Fig. 1. The observable band on the western blot was present only for the interaction between the antibody and Aβ_1–42_, and not for the other Aβ species. These results demonstrate the high selectivity of the mouse anti-Aβ_1–42_ primary antibody toward Aβ_1–42_.

In this Western blot experiment in Fig. 1, high-purity, commercially available Aβ_1–42_ peptide (Sigma-Aldrich, Cat. No. A9810) is used. Theoretically, Aβ_1–42_ peptide has a molecular weight of approximately 4.5 kDa. The observable band in Fig. 1 is clearly positioned below the 10 kDa marker. Due to the resolution limits by a 10% SDS-PAGE gel for very small peptides, the migration distance between 4 kDa and 10 kDa can be compressed. That is why the observable band of Aβ_1–42_ is so close to the 10 kDa marker.

### Titers of the anti-Aβ_1–42_ antibody

**Fig. 2:**
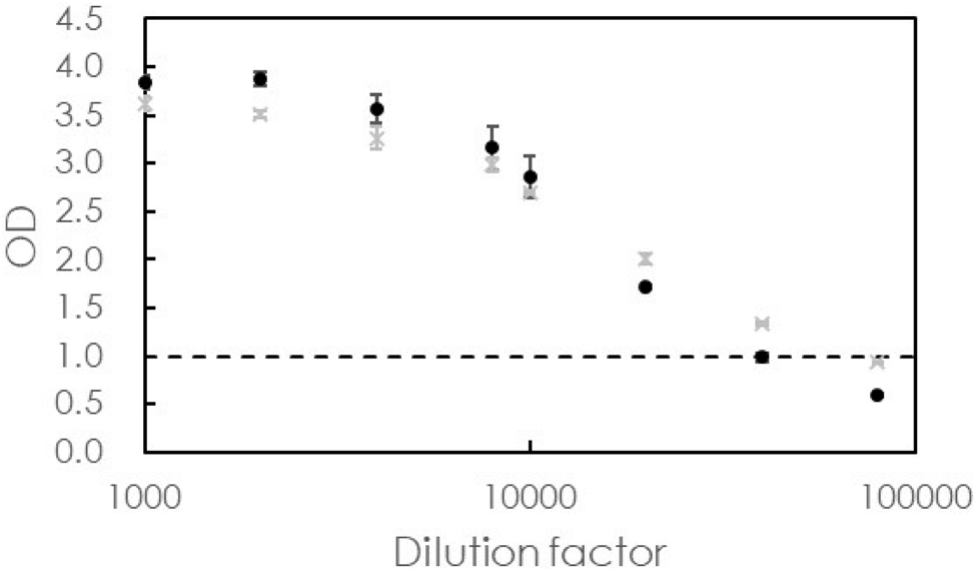
Determination of antibody titers *via* indirect ELISA. The graph shows the optical density (OD) values across a series of sample dilutions (ranging from 1 : 1000 to 1 : 80 000). Black circles (•) and grey crosses (×) represent two batches of synthesized antibody. The dashed horizontal line indicates the cutoff value (OD = 1.0) used to determine the antibody titer. Data are presented as mean ± SD (*n* = 3).

The optical density (OD) as a function of dilution factor for 1 mg mL^−1^ antibody is plotted in Fig. 2. The titers of two batches of synthesized antibody were characterized, as plotted with black and gray dots in Fig. 2, respectively. The error bars in Fig. 2 are contributed from duplicate measurements for a given dilution factor. The dilution factor-dependent ODs of the two batches of synthesized antibody almost overlap with each other. This reveals the batch-to-batch consistency in synthesizing the antibody.

In Fig. 2, the antibody, at dilution factors less than 2000, exhibits high and saturated values of OD (∼3.7). As the dilution factor increases from 2000 to 80 000, the OD gradually decreases from 3.7 to 0.8. Notably, the antibody showed a high titer, *i.e.* positive signals (OD > 1), at the dilution factor of 1 : 40 000. These results demonstrate the high titer of the antibody against Aβ_1–42_.

### Analytical curve of assaying Aβ_1–42_

**Fig. 3:**
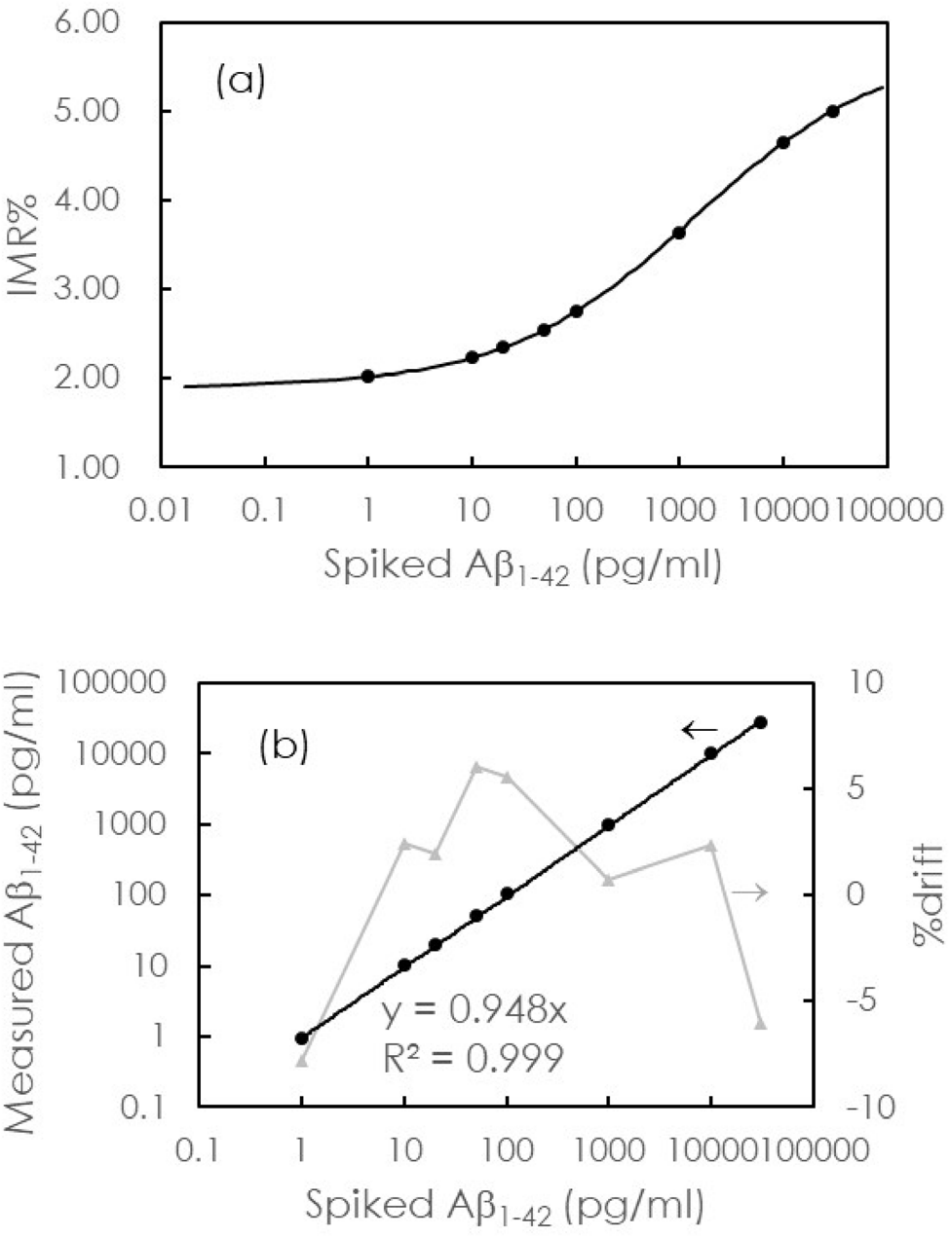
Aβ_1–42_ concentration-dependent (a) decreased magnetic susceptibility of the reagent (IMR%), and (b) measured Aβ_1–42_ concentrations plotted with black dots and deviations of the measured Aβ_1–42_ concentrations (% drift) plotted with gray triangles and the solid line. The solid line in (a) is the fitted logistic curve expressed as eqn (1).

Spiked Aβ_1–42_ (A1075, Sigma) solutions from 1 to 30 000 pg mL^−1^ were prepared for IMR measurements. The decreased magnetic susceptibility of the reagent, denoted as IMR%, was plotted as a function of the spiked Aβ_1–42_ concentration, as shown by the dots in Fig. 3(a). The experimental spiked Aβ_1–42_ concentration-dependent IMR% follows the four-parameter logistic function expressed as follows:1
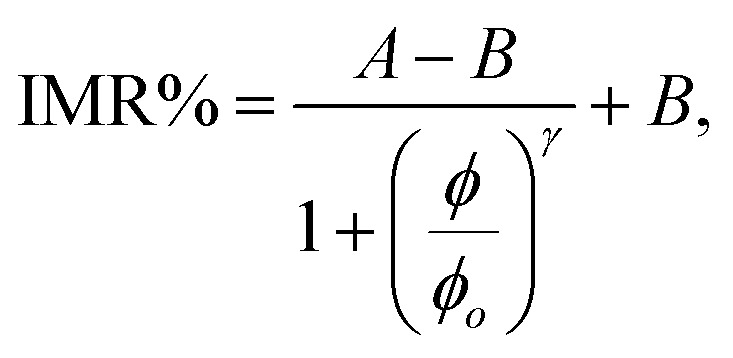
where *A*, *B*, *ϕ*_o_ and *γ* are fitting parameters. *ϕ* denotes the spiked Aβ_1–42_ concentration in pg ml^−1^. By fitting the data points in Fig. 3(a) to eqn (1), *A*, *B*, *ϕ*_o_ and *γ* are determined to be 1.90, 5.75, 1444.3 and 0.475, respectively. The fitted logistic curve is plotted as a solid line in Fig. 3(a). The coefficient of determination *R*^2^ is 0.999.

### Lower limit for detecting Aβ_1–42_

The parameter A in eqn (1) denotes the background level of IMR%, that is, the IMR% when the Aβ_1–42_ concentration is zero. The lower limit for detecting Aβ_1–42_ can be evaluated using the 3-σ criterion, in which the lower limit for detection (LLoD) is calculated as follows:

2 LLoD = Aβ_1–42_ concentration at an IMR% of *A* + 3*σ*,

where *σ* is the standard deviation of IMR% in duplicated IMR measurements at low Aβ_1–42_ concentrations (1 pg mL^−1^). According to the experimental results, the value of *σ* was found to be 0.0058, resulting in 1.92 for *A* + 3*σ*. The Aβ_1–42_ concentration at the IMR% of 1.92 was calculated to be 0.017 by using eqn (1). Therefore, the LLoD for assaying Aβ_1–42_ is 0.017 pg mL^−1^ (*i.e.*, 17 fg mL^−1^).

### Upper limit for detecting Aβ_1–42_

The measured IMR% values used for establishing the analytical curve with spiked Aβ_1–42_ concentrations from 1 to 30 000 pg mL^−1^ are converted to the measured Aβ_1–42_ concentrations using eqn (1). The relationships between the measured Aβ_1–42_ concentrations and the spiked Aβ_1–42_ concentrations were analyzed and plotted as black dots in Fig. 3(b). The relationship was found to be proportional, as indicated by the black solid line in Fig. 3(b). The coefficient of determination *R*^2^ between the dots and solid line was 0.999. The slope of the proportional relationship was 0.948.

Furthermore, the deviations of the measured Aβ_1–42_ concentrations from the spiked Aβ_1–42_ concentrations were analyzed as follows:3
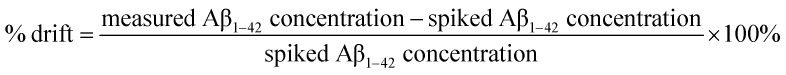


The values of % drift for the spiked Aβ_1–42_ concentrations ranged from −7.79% to 6.07%, plotted as gray dots in Fig. 3(b). The acceptable values of % drift were within the range from −10% to 10%. The calculated % drift values from 1 to 30 000 pg mL^−1^ Aβ_1–42_ shown in Fig. 3(b) were acceptable. The results shown in Fig. 3(b) demonstrate the validity of the proportionality between the measured and spiked concentrations, with the deviations becoming evident as the Aβ_1–42_ concentration approaches 30 000 pg mL^−1^. Thus, the high limit of detection for Aβ_1–42_ was 30 000 pg mL^−1^. A careful inspection of the results in Fig. 3(a) revealed that the Hook effect does not occur at 30 000 pg mL^−1^. This implies that the high limit of detection for Aβ_1–42_ could be greater than 30 000 pg mL^−1^.

### Interference tests of Aβ species on Aβ_1–42_

According to published papers, the measured concentrations of plasma Aβ_1–42_ in normal controls, aMCI patients and eADDs range from 10 to 50 pg mL^−1^.^22–26^ Hence, the Aβ_1–42_ samples were prepared by spiking 1, 16 and 100 pg mL^−1^ Aβ_1–42_ into plasma substitutes (Gelofusine; B Braun). On the other hand, the Aβ_1–40_ in human plasma was found to be several tens of pg mL^−1^, while other Aβ species in human plasma were several pg mL^−1^ or lower.^27–30^ Thus, the concentrations of interfering Aβ species were chosen to be in the range from 1 to 100 pg mL^−1^. In the interference tests, for an Aβ_1–42_ sample, interfering Aβ species included 2 pg mL^−1^ Aβ_3–42_, 1 pg mL^−1^ Aβ_p3–42_, 1 pg mL^−1^ Aβ_1–55_, 40 pg mL^−1^ Aβ_1–38_, 2 pg mL^−1^ Aβ_3–40_ and 100 pg mL^−1^ Aβ_1–40_. The measured concentrations of the Aβ_1–42_ samples without or with interfering Aβ species are listed in Table 1. The interference effect on assaying Aβ_1–42_ is quantified by calculating the recovery rate as follows:4
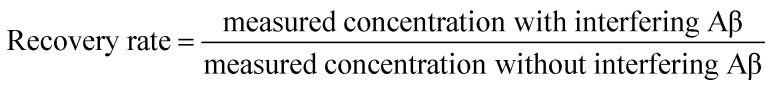


**Table 1:** Measured concentrations and recovery rates of Aβ_1–42_ assayed in samples without or with interference from Aβ using IMR

Interfering Aβ	Measured Aβ_1–42_ (pg mL^−1^)/recovery rate	Measured Aβ_1–42_ (pg mL^−1^)/recovery rate	Measured Aβ_1–42_ (pg mL^−1^)/recovery rate
None	0.99	16.46	102.5
Aβ_3–42_	0.98/0.990	16.67/1.013	102.0/0.995
Aβ_p3–42_	1.00/1.010	16.48/1.001	103.2/1.007
Aβ_1–55_	1.02/1.030	16.30/0.990	102.2/0.997
Aβ_1–38_	1.02/1.030	16.30/0.990	102.7/1.002
Aβ_3–40_	0.99/1.000	16.27/0.998	100.8/0.983
Aβ_1–40_	0.98/0.990	16.50/1.002	101.1/0.986

The recovery rates calculated using eqn (4) are listed in Table 1. Obviously, the recovery rates of the Aβ_1–42_ samples from 1 to 100 pg mL^−1^ were between 0.990 and 1.030. The acceptable recovery rates were within the range of 0.9 to 1.1, which means that there was no significant interference from other interfering Aβ species in assaying Aβ_1–42_. Thus, other Aβ species do not significantly contribute to the measurements of Aβ_1–42_ using IMR.

### Storage stability of the Aβ_1–42_ IMR reagent

A fresh batch of IMR Aβ_1–42_ reagent was aliquoted. One of the aliquots was immediately used for assaying Aβ_1–42_ in a PBS solution spiked with 50 pg mL^−1^ Aβ_1–42_, while the remaining aliquots were stored at 2–8 °C. After 24 h, an aliquot was thawed to room temperature for assaying Aβ_1–42_ in a 50 pg mL^−1^ Aβ_1–42_ PBS solution on the subsequent day. This procedure was repeated for all remaining aliquots on successive days. The measured Aβ_1–42_ concentrations on the first and other days are listed in Table 2. The drift of the measured Aβ_1–42_ concentration on the other day with respect to that on the first day is calculated as follows:5
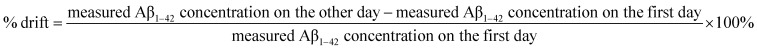


**Table 2:** Variation in the measured Aβ_1–42_ concentrations in the Aβ_1–42_ phosphate-buffered saline (PBS) solution measured on different days using aliquoted reagent stored at 2–8 °C before measurements

Measurement date	Storage period (day)	Measured concentration (pg mL^−1^)	% Drift
Dec. 25, 2018	0	50.97	—
Feb. 22, 2019	59	50.75	−0.42%
Apr. 17, 2019	113	50.23	−1.46%
May 30, 2019	156	50.80	−0.32%
Oct. 8, 2019	287	50.62	−0.68%
Mar. 19, 2020	440	50.02	−1.85%

The values of % drift on the other days are listed in Table 2.

The % drift ranged from −1.85% to −0.32% over 440 days. An acceptable %drift is −10% to 10%. Thus, there is no significant difference in the measured Aβ_1–42_ during 440 days of storage at 2–8 °C. The storage stability of the IMR Aβ_1–42_ reagent at 2–8 °C was at least 440 days.

## Discussion

The antibody of Aβ_1–42_ used in the immunomagnetic reduction (IMR) assay targets the 37-42 amino acid residues of Aβ_1–42_. Once amino acids 37-42 are partially lost or are structurally deformed, the association between the antibody and the peptides is broken. Aβ_1–40_ and Aβ_3–40_ lack amino acids 41 and 42, and Aβ_1–38_ lacks amino acids 39–42. As a result, the antibody cannot firmly bind with these peptides, as demonstrated in Fig. 1. The structure of the 37–42 amino acid residues is deformed in Aβ_1–55_, Aβ_3–42_ and Aβ_p3–42_ as compared with Aβ_1–42_.^31^ Hence, the antibody cannot firmly bind with Aβ_1–55_, Aβ_3–42_ or Aβ_p3–42_ either. Therefore, the antibody used in the IMR Aβ_1–42_ assay specifically targets Aβ_1–42_, as evidenced in Fig. 1.

The lower limit of detection for Aβ_1–42_ using IMR was found to be 17 fg mL^−1^, which is much lower than the published concentrations of Aβ_1–42_ in human plasma (10–50 pg mL^−1^) assayed with IMR.^22–26^ This reveals the ultrasensitivity of the IMR technology. At present, several analytical platforms, such as magnetic particle-based chemiluminescent enzyme immunoassay (MP-CLEIA), electrochemiluminescence (ECL), high-sensitivity chemiluminescence enzyme immunoassay (HISCL), single-molecule assays (SIMOA), single-molecule counting (SMC), and immunoprecipitation mass spectrometry (IP/MS), are available for assaying Aβ_1–42_ in human plasma in research or clinical practice. The reported low limits of detection (LLoDs) for these Aβ_1–42_ detection platforms are listed in Table 3.^32–37^ The LLoDs are several tens or hundreds of fg mL^−1^. Notably, IMR shows the lowest LLoD among the technologies.

**Table 3:** Low limit of detection (LLoD) of assaying Aβ_1–42_ using various technologies^26–31^^a^

Technology	MP-CLEIA	ECL	HISCL	IMR	SIMOA	SMC	IP/MS
LLoD (fg mL^−1^)	370	516	130	17	45	980	86

a MP-CLEIA: magnetic particle-based chemiluminescent enzyme immunoassay, ECL: electrochemiluminescence, HISCL: high-sensitivity chemiluminescence enzyme immunoassay, IMR: immunomagnetic reduction, SIMOA: single molecule array, SMC: single molecule counting, IP/MS: immunoprecipitation mass spectrometry.

In addition to the high-affinity antibody demonstrated in Fig. 2, four causative factors contribute to the ultrasensitive feature of IMR. The first factor is the homogeneously dispersed substrates of antibody-functionalized magnetic nanoparticles in solution. The second factor is the magnetic sensing of the high-transition-temperature superconducting quantum interference device (SQUID). The third factor is the high specificity of assaying the target antigen. The fourth factor is the low variation in duplicated measurements. The contributions of these factors to the ultrasensitive IMR assay are discussed individually.

The first causative factor is discussed. In an IMR reagent, the homogeneously dispersed magnetic nanoparticles provide a large area for the binding of antibodies to target biomolecules. The binding area between antibody and antigen in an IMR Aβ_1–42_ test is estimated to be approximately 600 mm^2^. The bottom area of a well in a 96-well plate is approximately 35 mm^2^. The binding area of the nanoparticles used in the IMR measurement was greater than that of a well in a 96-well plate by approximately 16.5 fold. The larger binding area enhanced the possibility of associations between the antibodies and target biomolecules, improving the ability to significantly detect target biomolecules at ultralow concentrations and resulting in the lower LLoD.

In the IMR assay, lower concentrations of target biomolecules reveal slight decreases in the magnetic susceptibility of the reagent. An ultrasensitive sensor able to detect the slight decreases in the magnetic susceptibility of the reagent is needed to achieve an ultrasensitive immunoassay. Körber *et al.* demonstrated that the SQUID is an extremely sensitive sensor for detecting tiny changes in the magnetic susceptibility of a unit of detection,^38^ which enables precise measurements of ultralow-concentrated biomolecules in IMR. This is the reason why the high-transition-temperature SQUID is a causative factor contributing to the ultrasensitive feature of IMR.

For conventional immunoassays, such as chemiluminescence enzyme immunoassays, electrochemiluminescence, and digital enzyme-linked immunosorbent assays (ELISAs), the analytical specificity of assaying biomolecules predominantly depends on the use of paired capture and detection antibodies. However, in IMR, only one type of primary antibody is used. IMR is a single-antibody immunoassay. To ensure the ultrahigh specificity of assaying Aβ_1–42_ in IMR measurements, the selection of the primary antibody is critical. The results shown in Fig. 1 demonstrate the high specificity of the primary antibody against Aβ_1–42_.

Moreover, the analytical specificity of IMR measurements was enhanced with the so-called spin-wash technology, explored by Yang *et al.* in 2010.^39^ The primary antibody immobilized on magnetic nanoparticles could bind nonspecific biomolecules. Moreover, the primary antibody on magnetic nanoparticles can associate with target biomolecules. The binding force between the primary antibody and target biomolecules is stronger than that between the primary antibody and nonspecific biomolecules. The bound biomolecules interact with centrifugal and shearing forces as the nanoparticles oscillate under external alternating current magnetic fields. Increasing the oscillating frequencies of nanoparticles by manipulating the frequencies of the external alternating current magnetic fields improves the centrifugal and shearing forces. At given specific frequencies, both the centrifugal and shearing forces are stronger than the binding force between the primary antibody and nonspecific biomolecules, but weaker than that between the primary antibody and target biomolecules, and nonspecific binding is suppressed. The decreased magnetic susceptibility of the reagent in IMR is primarily attributed to the specific interactions between primary antibodies and target biomolecules. Hence, the specificity of the primary antibody and the spin-wash technology contribute to the ultrahigh specificity of assaying Aβ_1–42_ in IMR measurements. Therefore, with high-specificity antibodies and spin-wash technology, IMR is an ultraspecific assay for Aβ_1–42_.

The LLoD is determined according to the 3*σ* criteria *via*eqn (2). The *σ* denotes the standard deviation of IMR% in duplicated IMR measurements at a low concentration, *i.e.* 1 pg mL^−1^. This implies that the variations in duplicated IMR measurements are dominant over the LLoD. The variations in the measured signals, IMR%, and the measured Aβ_1–42_ concentrations converted *via*eqn (1) for spiked Aβ_1–42_ solutions of various concentrations from 1 to 100 pg mL^−1^ in the duplicated IMR measurements are listed in Table 4. In the case of 1 pg mL^−1^, the average value, standard deviation, and coefficient of variation for IMR% were found to be 2.015, 0.0058 and 0.29%, respectively. The average value, standard deviation and coefficient of variation for the measured Aβ_1–42_ concentrations were obtained to be 0.922 pg mL^−1^, 0.103 pg mL^−1^ and 11.13%, respectively. Remarkably, the coefficients of variation in the case of the spiked 1 pg mL^−1^ Aβ_1–42_ solution are obviously lower (0.29% for the measured IMR% and 11.13% for the measured Aβ_1–42_ concentration). For other spiked concentrations of Aβ_1–42_, the coefficients of variation are less than 1% for the measured IMR% and 10% for the measured Aβ_1–42_. These results demonstrate the low variations in IMR measurements, attributed to the low value for *σ* in eqn (2), resulting in ultrahigh sensitivity for the IMR Aβ_1–42_ assay.

**Table 4:** Measured signals (IMR%) and concentrations in duplicated measurements of the IMR assay for Aβ1-42 of various spiked concentrations from 1 to 100 pg mL^−1^. Measured Aβ1-42 concentrations are converted from measured IMR% *via*eqn (1)^a^

Spiked Aβ_1–42_ (pg mL^−1^)	Measured IMR%					Measured Aβ_1–42_ (pg mL^−1^)				
	1st	2nd	Mean	SD	CV%	1st	2nd	Mean	SD	CV%
1	2.019	2.011	2.015	0.0058	0.29	0.995	0.850	0.922	0.103	11.13
10	2.229	2.243	2.236	0.0100	0.45	9.740	10.74	10.24	0.709	6.92
20	2.350	2.351	2.350	0.0012	0.05	20.30	20.49	20.40	0.134	0.66
50	2.558	2.569	2.564	0.0079	0.31	51.90	54.17	53.04	1.603	3.02
100	2.776	2.748	2.762	0.0200	0.72	110.3	100.9	105.6	6.648	6.30

a 1^st^: the first measurement, 2nd: the second measurement, mean: average value of the 1st and the 2nd measurements, SD: standard deviation of the 1st and the 2nd measurements, CV%: coefficient of variation calculated *via* SD/mean × 100%.

Chen *et al.* revealed that aggregated Aβ_1–42,_ such as oligomers and protofibrils, play a role in the development of AD.^40^ Therapeutic antibodies developed by Biogen and Eisai target soluble aggregated Aβ_1–42_ in the brain.^41^ Thus, it is important to be able to assay aggregated Aβ_1–42_ in human plasma. Using single-antibody technology such as IMR and sandwich technology such as SIMOA, Li *et al.* investigated the measured conformations of Aβ_1–42_.^42^ The results clearly demonstrated that the sandwich technique can be used to detect monomers of Aβ_1–42_, whereas the single-antibody technique can be used to detect monomers and aggregated forms of Aβ_1–42_. As a result, the correlations between the Aβ_1–42_ levels measured by these two platforms were limited.

Li *et al.* further compared the differences between normal controls and AD patients by assaying plasma Aβ_1–42_ using single-antibody technology (IMR) and sandwich technology (SIMOA).^42^ A clear difference in the measured plasma levels of Aβ_1–42_ was detected using single-antibody technology (IMR). Moreover, increased plasma Aβ_1–42_ levels were observed in AD patients compared with those in normal controls. These results are attributed to the detection of aggregated Aβ_1–42_ in plasma with IMR.

The increase in plasma Aβ_1–42_ in aMCI patients or eADDs detected through IMR was reported by Li *et al.* and other groups.^22–26^ Chao *et al.* reviewed studies on plasma Aβ_1–42_ assayed with IMR in 24 cognitively unimpaired (CU) cohorts, 16 aMCI cohorts, and 23 eADD cohorts published between 2012 and 2023.^43^ The reported mean values of plasma Aβ_1–42_ ranged from 14.6 to 16.9 pg mL^−1^ in the CU group, from 15.7 to 18.6 pg mL^−1^ in the aMCI group and from 16.4 to 34.2 pg mL^−1^ in the eADD group. The level of plasma Aβ_1–42_ assayed with IMR increased significantly from the CU group to the aMCI group (*p* < 0.0001), but remained almost unchanged from the aMCI group to the eADD group (*p* > 0.05). Notably, Lue *et al.* and Hu *et al.* reported the age (20–90 years) and sex independence of plasma Aβ_1–42_ assayed with IMR in the CU group.^44,45^ Hence, the elevated plasma Aβ_1–42_ levels in aMCI and eADD patients, compared with those in CU patients, are predominantly attributable to the disease. Collectively, the consistent and significant increases in plasma Aβ_1–42_ levels measured by IMR support its clinical utility for the assessment and diagnosis of early Alzheimer's disease and related cognitive disorders.

## Conclusion

Although only a single primary antibody targeting the C-terminal domain (37–42 amino acid residues) of Aβ_1–42_ is used in the immunomagnetic reduction (IMR) assay, the high specificity of detecting Aβ_1–42_ is demonstrated. The interference effect of Aβ_3–42_, pyroglutamate-modulated Aβ_3–42_ (Aβ_p3–42_), Aβ_1–55_, Aβ_1–38_, Aβ_3–40,_ and Aβ_1–40_ is not significant in the Aβ_1–42_ assay. Thus, the IMR Aβ_1–42_ assay specifically detects Aβ_1–42_, and neither Aβ_*x*–42_ nor Aβ_1–*y*_ species. The high specificity and spin-wash technology contribute to the ultrahigh specificity of the IMR assay. In addition, the lower and upper limits of detection of the IMR Aβ_1–42_ assay were 17 fg mL^−1^ and 30 000 pg mL^−1^, respectively. This broad dynamic range provides sufficient sensitivity and robustness for precise quantification of Aβ_1–42_ in human plasma.

## Conflicts of interest

All authors are employees of MagQu Co., Ltd, and S. Y. Yang is a shareholder of MagQu Co., Ltd.

## Data Availability

The original contributions presented in this study are included in the article. Further inquiries can be directed to the corresponding author.
